# Association of chronic and acute inflammation of the mucosa-associated lymphoid tissue with psychiatric disorders and suicidal behavior

**DOI:** 10.1038/s41398-019-0568-5

**Published:** 2019-09-12

**Authors:** Josef Isung, Kayoko Isomura, Catarina Almqvist, Paul Lichtenstein, Henrik Larsson, Tomas Wester, Christian Rück, Lorena Fernández de la Cruz, Anna Sidorchuk, David Mataix-Cols

**Affiliations:** 1 Centre for Psychiatry Research, Department of Clinical Neuroscience, Karolinska Institutet & Stockholm Health Care Services, Stockholm County Council, Stockholm, Sweden; 2 Department of Medical Epidemiology and Biostatistics, Karolinska Institutet, Stockholm, Sweden; 3 Astrid Lindgren Children’s Hospital, Karolinska University Hospital, Stockholm, Sweden; 4 School of Medical Sciences, Örebro University, Örebro, Sweden; 5 Division of Pediatric Surgery, Department of Women’s and Children’s Health, Karolinska Institutet, Stockholm, Sweden

**Keywords:** Physiology, Psychiatric disorders

## Abstract

Immune dysregulation due to chronic inflammation is a hypothesized risk factor underlying psychiatric disorders and suicidal behavior. Whether tonsillectomy and acute appendicitis used, respectively, as proxies for chronic and acute inflammation within the mucosa-associated lymphoid tissue (MALT) are associated with psychiatric disorders and suicidal behavior is currently unknown. A birth cohort study was conducted including 3,052,875 individuals born in Sweden between 1973 and 2003. We identified 210,686 individuals ever exposed to tonsillectomy and 86,928 individuals ever exposed to acute appendicitis, as well as 317,214 clusters of siblings discordant for tonsillectomy, and 160,079 sibling clusters discordant for acute appendicitis. Outcomes were an aggregate risk of ‘any psychiatric disorder’, ‘any suicidal behavior’, 12 individual psychiatric disorders, suicide attempts and deaths by suicide. Tonsillectomy was associated with increased odds of ‘any psychiatric disorder’ (adjusted odds ratio [aOR] = 1.39; 95% confidence interval (CI) = 1.38–1.41) and ‘any suicidal behavior’ (aOR = 1.41; 95% CI = 1.37–1.44), and most individual disorders. Acute appendicitis also increased the odds of ‘any psychiatric disorder’ and ‘any suicidal behavior’ (aOR = 1.23; 95% CI = 1.20–1.25, and aOR = 1.32; 95% CI = 1.28–1.37, respectively). Exposure to both tonsillectomy and appendicitis was associated with the highest odds of ‘any psychiatric disorder’ (aOR = 1.70; 95% CI = 1.59–1.82) and ‘any suicidal behavior’ (aOR = 1.90; 95% CI = 1.70–2.12). In sibling comparisons, the associations were attenuated but remained significant. We conclude that inflammation within the MALT, particularly when chronic, is robustly associated with a broad range of psychiatric disorders and suicidal behavior.

## Introduction

The concept of chronic inflammation with an ensuing immune dysregulation has been suggested as a key pathophysiological risk factor for several psychiatric disorders, including depression^1^, autism spectrum disorders (ASD)^2^, schizophrenia^3^, bipolar disorder^4^, obsessive-compulsive disorder (OCD)^5^, Tourette’s and chronic tic disorder (TD)^5^, or anorexia nervosa^6^, and suicidal behavior^7,8^. Repeated or persistent inflammatory insults during vulnerable developmental phases have been shown to impact the developing brain^9^. At a cell level, chronic inflammation primes immune cells into phenotypes that can react in a detrimental way upon subsequent insults on the immune system, suggesting mechanisms that could explain these brain–immune system interactions^1,10^.

The tonsils and the caecal appendix are secondary lymphoid organs that are part of the mucosa-associated lymphoid tissue (MALT)^11–13^. The tonsils are recognized as having unique immunological properties, implicated in regulation of T-cell function and extrathymic T-cell development, making them relevant for immunological self-tolerance^14,15^. The caecal appendix is thought to act as a reservoir for the colonic flora, with the ability to restore a healthy microbiome after infections^16^. Components of the MALT represent a first-line barrier under constant exposure to foreign pathogens and inflammatory challenges. Common infectious and inflammatory conditions in the MALT include tonsillitis, hypertrophy of the glands, and appendicitis, among others^11–13,17^. Recurrent tonsillitis, obstructive hypertrophy of the glands, and obstructive sleep apnea, suggesting chronic inflammation as the underlying pathological process, are common indications for tonsillectomy^18^. Acute appendicitis, also a common condition that in a majority of cases leads to appendectomy^19^, is generally regarded as an acute inflammatory process^20^. Pathology within these organs is highly prevalent; in Sweden alone, about 10,000 tonsillectomies^21^ and 10,000 appendectomies^22^ are performed yearly. These medical interventions occur mainly during childhood and adolescence, which constitutes the time frame when these organs are most physiologically active^21,23–25^, and also a time of onset for most psychiatric disorders^26,27^.

Both tonsillectomy and appendectomy are associated with a range of adverse health outcomes, presumably due to alterations of the immune system, which are not fully understood. For example, both interventions have been associated with a subsequent risk of acute myocardial infarction^23^. Tonsillectomies have also been associated with an overall increased risk of autoimmune diseases^24^, as well as risk of respiratory, allergic, and infectious diseases^28^, whereas for appendectomies, a protective effect has been reported for ulcerative colitis^25,29^ and Parkinson’s disease^30^. Whether these interventions, as well as the underlying inflammatory processes of the MALT, are also (positively or negatively) associated with psychiatric disorders is still unknown.

In the current study, we aimed to extend the existing knowledge regarding the complex pathophysiological relationship between inflammation, psychiatric disorders, and suicidal behavior. Capitalizing on the extensive nationwide registers in Sweden, which provide virtually complete information on all medical diagnoses and familial pedigrees, we conducted a population-based study to elucidate whether tonsillectomies and acute appendicitis (as proxies for chronic and acute inflammation of the MALT, respectively), are associated with psychiatric disorders and suicidal behavior while controlling for familial confounders, parental psychopathology, and history of suicidal behavior, among other variables.

## Materials and methods

The study was approved by the Regional Ethical Review Board in Stockholm (reference 2013/862-31/5). The requirement for informed consent was waived because the study was register-based and the included individuals were not identifiable at any time.

### Data sources

Using the unique Swedish national identification number^31^, we linked several Swedish nationwide health and administrative registers. Demographic and migration data were extracted from the Swedish Total Population Register and the Migration Register^32^, respectively. The kinship was identified from the Multi-Generation Register^33^, and information on the causes and dates of death was collected from the Cause of Death Register^34^. The Prescribed Drug Register (PDR) covered all prescribed and dispensed medications since July 2005^35^. The National Patient Register (NPR) provided information on diagnoses given in both inpatient (from 1969, with nationwide coverage for psychiatric disorders from 1973) and outpatient specialist services (since 2001) in Sweden^36^. These diagnoses are based on the International Classification of Diseases (ICD) in its eighth (ICD-8; 1969–1986), ninth (ICD-9; 1987–1996), and tenth (ICD-10; 1997–2013) revisions.

### Study population

We used a longitudinal, population-based, birth cohort design. The cohort consisted of all singleton live births in Sweden from January 1, 1973 through December 31, 2003 with two biological parents identifiable from the Multi-Generation Register. The study population was followed up until December 31, 2013. To control for familial confounding, each individual was linked to their full siblings using the Multi-Generation Register. To link the relatives, a family identification number was created. Siblings were identified within the same birth cohort (i.e., 1973–2003) and were considered for the family level analysis if they had at least one full sibling discordant for the exposure in question, separately for tonsillectomies and acute appendicitis.

### Variables

#### Exposures

Individuals with a record of tonsillectomy or acute appendicitis between 1973 and 2013 were identified from the NPR (Supplementary Table S1) and considered exposed. The corresponding national operational codes and ICD codes were retrieved along with information on age of exposure. For additional analyses, we constructed a variable for the joint exposure to tonsillectomy and acute appendicitis which was categorized as: tonsillectomy only, acute appendicitis only, tonsillectomy plus acute appendicitis, or none.

#### Outcomes

A lifetime record of a psychiatric disorder or suicidal behavior constituted the outcomes. Psychiatric disorders included OCD, TD, attention-deficit/hyperactivity disorder (ADHD), ASD, schizophrenia and other psychotic disorders, bipolar disorder, major depression disorder and other mood disorders, generalized anxiety disorder, agoraphobia, social anxiety disorder, anorexia nervosa, and substance use disorders (Supplementary Table S2). To avoid diagnostic misclassification, we set a minimal age at what the individuals with lifetime diagnoses of the specific psychiatric disorders could be defined as cases (Supplementary Table S2). In addition, we created a combined ‘any psychiatric disorder’ variable.

For suicidal behavior outcomes, we identified all deaths by suicide (through the Cause of Death Register) and all records of lifetime suicide attempts (through the NPR records of inpatient and specialized outpatient care) (Supplementary Table S3), if recorded at age 10 years or above. In addition, a combined ‘any suicidal behavior’ variable was created.

#### Covariates

Information on birth year and sex were collected to be used in adjusted models. The following variables were also considered as potential confounders: county of birth (to control for differences in medical routines between areas with different level of urbanicity), maternal and paternal age at childbirth (5-year increments from the age of 20 years)^37^, highest parental education level^38^, number of siblings, parental lifetime history of psychiatric disorders, and parental history of suicidal behavior (the latter two variables were categorized as ‘ever’, if at least one parent had any of the corresponding records or ‘never’).

### Data analysis

#### Individual data analysis

Logistic regression models were fitted to estimate the associations, separately for tonsillectomy and acute appendicitis, with the following outcomes: (i) ‘any psychiatric disorder’, (ii) specific psychiatric disorders, (iii) ‘any suicidal behavior’, (iv) suicide attempts, and (v) deaths by suicide. Odds ratio (OR) with corresponding 95% confidence intervals (CI) was used to report the results. A model minimally adjusted for year of birth and sex was followed by a model additionally adjusted for all above-mentioned covariates.

#### Sibling analysis

Logistic regression was fitted in the subsample of full siblings to compare siblings discordant for the exposure (separately for tonsillectomy and acute appendicitis). This comparison accounts for unmeasured familial confounders given that full siblings share on average 50% of genetic factors and much of the early environment. The model further adjusted for year of birth and sex of both exposed and unexposed siblings.

#### Additional analyses

In order to assess the association between joint exposure to tonsillectomy and acute appendicitis and psychiatric disorders or suicidal behaviors, we conducted a separate analysis among the individuals exposed to only tonsillectomies, only appendicitis, or both exposures, in comparison to those with none of the exposures.

In order to explore whether chronic or acute MALT inflammation increased the risk of *subsequent* development of outcomes, we fitted a Poisson regression model to estimate incidence rate ratio (IRR) and 95% CI as a measure of relative risk. These analyses were conducted separately for exposure to tonsillectomy and exposure to appendicitis. All individuals exposed from birth to age 9 years were compared with individuals free from exposure at any age and the whole cohort was followed from age 10 years for the incident cases of psychiatric disorders and suicidal behavior. The participants were excluded from the analysis if they had the outcome before age 10. The same analysis was repeated for age of exposure from birth to age 19 years with follow-up for an outcome record starting from age 20 years. Individuals with an outcome record before age 20 years were excluded. The rationale to start the follow-up for incident cases of outcomes from the age of 10 years and the age of 20 years, respectively, was based on descriptions of the tonsils and the caecal appendix being mostly physiologically active during childhood and adolescence^16,39^, and in line with previous studies^24,25^.

The main analyses were repeated for all study outcomes excluding individuals with an outcome diagnosed before 2001 (i.e., when the NPR records were solely based on inpatient visits). Given the availability of data from both inpatient and outpatient care in 2001 onward, this analysis was aimed to address a potential bias from more severe cases and ensure generalizability of the results to patients with less severe psychiatric illness or suicide attempts.

To account for non-independence between repeated observations within families, we clustered all analyses by family identification number and used a robust sandwich estimator of standard errors. Data management was performed with SAS, version 9.4 (SAS Institute, Irvine, CA), and analyses were performed using STATA, version 15.1 (StataCorp LLC, College Station, TX).

## Results

In our cohort we identified 210,686 individuals (50.16% women; *n* = 105,687) with a record of tonsillectomy and 86,928 individuals (44.05% women; *n* = 38,291) with a record of acute appendicitis. Table 1 reports the descriptive characteristics of the total cohort and the full sibling subcohorts by exposure status.

**Table 1:** Descriptive characteristics of the total cohort and full siblings subcohorts

	Tonsillectomy, %				Acute appendicitis, %			
	Whole cohort		Full siblings		Whole cohort		Full siblings	
	Exposed (*n* = 210,686)	Unexposed (*n* = 2,842,189)	Exposed (*n* = 135,524)	Unexposed (*n* = 181,690)	Exposed (*n* = 86,928)	Unexposed (*n* = 2,965,947)	Exposed (*n* = 64,386)	Unexposed (*n* = 95,693)
*Sex*								
Women	50.16	48.48	50.29	48.30	44.05	48.73	43.78	48.68
*Maternal age at childbirth*								
<20 years	4.06	3.41	3.17	3.61	3.90	3.45	3.19	3.18
20–24 years	25.80	22.34	26.03	26.44	24.56	22.52	25.26	23.88
25–29 years	36.97	36.63	38.93	37.35	36.97	36.64	38.73	36.86
30–34 years	23.32	26.06	23.40	23.56	24.24	25.92	23.97	25.29
35–39 years	8.34	9.78	7.41	7.92	8.78	9.71	7.77	9.34
40–44 years	1.44	1.71	1.02	1.09	1.51	1.70	1.05	1.41
≥45 years	0.06	0.07	0.04	0.03	0.05	0.07	0.03	0.05
*Paternal age at childbirth*								
<20 years	0.90	0.76	0.55	0.64	0.88	0.77	0.56	0.60
20–24 years	13.90	11.72	12.98	13.31	13.27	11.83	12.88	12.01
25–29 years	33.55	31.83	34.97	34.02	33.60	31.90	35.02	32.55
30–34 years	29.53	31.27	30.62	30.16	29.96	31.18	30.75	31.08
35–39 years	14.62	16.03	14.48	14.95	14.82	15.96	14.47	16.11
40–44 years	5.22	5.82	4.61	5.02	5.19	5.79	4.57	5.53
≥45 years	2.28	2.58	1.79	1.89	2.27	2.57	1.76	2.11
*Parental education* ^a^								
Elementary (≤9 years)	1.44	1.85	1.12	1.26	1.91	1.82	1.21	1.27
Secondary (10–12 years)	36.00	31.60	35.20	36.07	34.39	31.83	32.93	33.82
Higher (≥13 years)	62.50	65.71	63.63	62.62	63.63	65.54	65.83	64.86
Unknown	0.06	0.83	0.05	0.05	0.06	0.80	0.04	0.04
*Parental psychiatric disorders* ^a^								
Ever	21.58	18.16	18.83	19.05	19.60	18.36	17.47	17.58
*Parental suicidal behavior* ^a^								
Ever	8.76	7.01	7.46	7.56	7.85	7.11	6.88	6.91
*Age at exposure*								
0–9 years	57.73	na	55.56	na	15.94	na	15.08	na
10–19 years	31.09	na	32.97	na	46.93	na	49.61	na
≥20 years	11.17	na	11.47	na	37.13	na	35.31	na

*na* not applicable

^a^Full siblings share these parental characteristics; however, the proportions reported for exposed and unexposed siblings differ since ‘exposed to unexposed’ ratio is not 1:1

### Tonsillectomies

A total of 29,615 individuals who had tonsillectomies (14.06%) and 293,815 of the unexposed individuals (10.34%) had at least one diagnosis of psychiatric disorders (Table 2). In a fully adjusted model, individuals with tonsillectomy had a 39% higher likelihood of ‘any psychiatric disorder’ (aOR = 1.39 [95% CI 1.38–1.41]), compared with their unexposed counterparts. The likelihood of having a specific psychiatric disorder was also significantly higher for most outcomes with aORs ranging between 1.17 (social anxiety disorder) and 1.54 (ADHD). Tonsillectomies were not significantly associated with anorexia nervosa or schizophrenia. Exposed individuals also had an increased likelihood of ‘any suicidal behavior’ (aOR = 1.41 [95% CI 1.37–1.44]) and suicide attempts (aOR = 1.42 [95% CI 1.39–1.46]), compared with those without lifetime record of tonsillectomy (Table 2). The median number of years between the tonsillectomy and the psychiatric outcomes ranged between 5 and 13 years (Supplementary Table S4, Supplementary Figure S1). In the sibling cohort, the associations were attenuated, but remained significant for the combined outcomes and for most of the specific outcomes, except for OCD, schizophrenia, and anorexia nervosa (Table 2).

**Table 2:** Associations between exposure to tonsillectomy and psychiatric disorders and suicidal behavior in the total cohort and in the sibling analysis

	Whole cohort, no. (%)				Full siblings, no. (%)		
	Exposed (*n* = 210,686)	Unexposed (*n* = 2,842,189)	Minimally adjusted OR (95% CI)^a^	Fully adjusted OR (95% CI)^b^	Exposed (*n* = 135,524)	Unexposed (*n* = 181,690)	Minimally adjusted OR (95% CI)^c^
Any psychiatric disorder^d^	29,615 (14.06)	293,815 (10.34)	**1.46** (**1.44–1.47)**	**1.39** (**1.38–1.41)**	18,010 (13.29)	20,291 (11.17)	**1.22** (**1.20–1.25)**
Obsessive-compulsive disorder	1554 (0.74)	17,726 (0.62)	**1.22** (**1.16–1.29)**	**1.20** (**1.13–1.26)**	938 (0.69)	1177 (0.65)	1.07 (0.98–1.16)
Tourette’s and chronic tic disorders	614 (0.29)	4997 (0.18)	**1.53** (**1.41–1.67)**	**1.43** (**1.31–1.56)**	342 (0.25)	367 (0.20)	**1.24** (**1.07–1.43)**
Attention-deficit/hyperactivity disorder	10,852 (5.15)	85,154 (3.00)	**1.66** (**1.62–1.69)**	**1.54** (**1.51–1.57)**	6 109 (4.51)	6345 (3.49)	**1.30** (**1.25–1.34)**
Autism spectrum disorders	2044 (0.97)	17,935 (0.63)	**1.42** (**1.36–1.49)**	**1.37** (**1.31–1.44)**	1197 (0.88)	1236 (0.68)	**1.28** (**1.19–1.39)**
Schizophrenia and other psychotic disorders	1300 (0.62)	18,809 (0.66)	**1.10** (**1.04–1.17)**	1.06 (1.00–1.12)	802 (0.59)	1029 (0.57)	1.09 (1.00–1.20)
Bipolar disorder	1879 (0.89)	17,842 (0.63)	**1.56** (**1.49–1.64)**	**1.48** (**1.41–1.55)**	1143 (0.84)	1246 (0.69)	**1.22** (**1.13–1.32)**
Major depression disorder and other mood disorders	12,371 (5.87)	124,096 (4.37)	**1.46** (**1.44–1.49)**	**1.40** (**1.37–1.43)**	7 750 (5.72)	8554 (4.71)	**1.23** (**1.19–1.26)**
Generalized anxiety disorder	1419 (0.67)	15,717 (0.55)	**1.32** (**1.25–1.40)**	**1.28** (**1.22–1.36)**	885 (0.65)	1021 (0.56)	**1.16** (**1.06–1.27)**
Agoraphobia	524 (0.25)	5836 (0.21)	**1.35** (**1.23–1.47)**	**1.28** (**1.17–1.40)**	321 (0.24)	367 (0.20)	**1.18** (**1.02–1.37)**
Social anxiety disorder	1356 (0.64)	16,165 (0.57)	**1.21** (**1.15–1.28)**	**1.17** (**1.11–1.24)**	894 (0.66)	1003 (0.55)	**1.20** (**1.10–1.32)**
Anorexia nervosa	710 (0.34)	9815 (0.35)	0.95 (0.88–1.03)	0.97 (0.90–1.05)	481 (0.35)	661 (0.36)	0.94 (0.83–1.05)
Substance use disorders	10,073 (4.78)	102,528 (3.61)	**1.46** (**1.43–1.49)**	**1.38** (**1.35–1.41)**	6208 (4.58)	7078 (3.90)	**1.21** (**1.17–1.25)**
Any suicidal behavior^d^	7841 (3.72)	76,948 (2.71)	**1.50** (**1.46–1.53)**	**1.41** (**1.37–1.44)**	4892 (3.61)	5480 (3.02)	**1.22** (**1.17–1.26)**
Death by suicide	274 (0.13)	4116 (0.14)	1.13 (1.00–1.28)	1.04 (0.92–1.18)	169 (0.12)	246 (0.14)	1.01 (0.83–1.22)
Suicide attempt	7674 (3.64)	74,090 (2.61)	**1.51** (**1.48–1.55)**	**1.42** (**1.39–1.46)**	4789 (3.53)	5312 (2.92)	**1.23** (**1.18–1.27)**

Significant ORs are marked in bold

*OR* odds ratio, *CI* confidence interval

^a^Adjusted for individual’s year of birth and sex

^b^Additionally adjusted for county, maternal and paternal age at childbirth, parental highest educational level, parental lifetime history of psychiatric disorders, parental history of suicidal behavior, and number of siblings

^c^Adjusted for year of birth and sex on both exposed and unexposed siblings

^d^Total numbers and percentage of the specific outcomes may not sum up to that of the combined outcomes as the study participants may have more than one specific outcome

### Acute appendicitis

A total of 11,464 individuals who had acute appendicitis (13.19%) and 311,966 of the unexposed individuals (10.52%) had at least one diagnosis of a psychiatric disorder (Table 3). In fully adjusted models, acute appendicitis was associated with a 23% increased likelihood of ‘any psychiatric disorder’ (aOR = 1.23 [95% CI 1.20–1.25]) (Table 3). Similarly, the significant associations were observed for the majority of the specific psychiatric disorders with aORs ranging between 1.11 (OCD) and 1.35 (bipolar disorder). No significant associations were found for ASD, schizophrenia, and agoraphobia. Exposed individuals also had an increased likelihood of ‘any suicidal behavior’ (aOR = 1.32 [95% CI 1.28–1.37]) and suicide attempts (aOR = 1.35 [95% CI 1.30–1.39]), compared with those that were unexposed (Table 3). The median number of years between the acute appendicitis and the outcomes ranged between 0 and 8 years (Supplementary Table S4; Supplementary Figure S2). The associations were attenuated in the sibling comparison, but remained significant for the combined outcomes, as well as for some of the specific outcomes (Table 3).

**Table 3:** Associations between exposure to acute appendicitis and psychiatric disorders and suicidal behavior in the total cohort and in the sibling analysis

	Whole cohort, no. (%)				Full siblings, no. (%)		
	Exposed (*n* = 86,928)	Unexposed (*n* = 2,965,947)	Minimally adjusted OR (95% CI)^a^	Fully adjusted OR (95% CI)^b^	Exposed (*n* = 64,386)	Unexposed (*n* = 95,693)	Minimally adjusted OR (95% CI)^c^
Any psychiatric disorder^d^	11,464 (13.19)	311,966 (10.52)	**1.25** (**1.23–1.28)**	**1.23** (**1.20–1.25)**	7 995 (12.42)	10 732 (11.22)	**1.13** (**1.10–1.17)**
Obsessive-compulsive disorder	640 (0.74)	18,640 (0.63)	**1.13** (**1.04–1.22)**	**1.11** (**1.03–1.20)**	443 (0.69)	653 (0.68)	1.03 (0.91–1.16)
Tourette’s and chronic tic disorders	169 (0.19)	5442 (0.18)	**1.20** (**1.03–1.40)**	**1.18** (**1.01–1.38)**	119 (0.18)	131 (0.14)	**1.31** (**1.02–1.68)**
Attention-deficit/hyperactivity disorder	2998 (3.45)	93,008 (3.14)	**1.24** (**1.19–1.28)**	**1.21** (**1.16–1.26)**	2 046 (3.18)	2 730 (2.85)	**1.13** (**1.07–1.19)**
Autism spectrum disorders	466 (0.54)	19,513 (0.66)	0.95 (0.87–1.04)	0.93 (0.85–1.02)	333 (0.52)	551 (0.58)	0.90 (0.79–1.03)
Schizophrenia and other psychotic disorders	716 (0.82)	19,393 (0.65)	**1.09** (**1.01–1.17)**	1.06 (0.99–1.15)	466 (0.72)	630 (0.66)	1.07 (0.95–1.21)
Bipolar disorder	840 (0.97)	18,881 (0.64)	**1.39** (**1.30–1.49)**	**1.35** (**1.26–1.45)**	567 (0.88)	708 (0.74)	**1.22** (**1.09–1.36)**
Major depression disorder and other mood disorders	5464 (6.29)	131,003 (4.42)	**1.36** (**1.32–1.40)**	**1.33** (**1.29–1.37)**	3827 (5.94)	4938 (5.16)	**1.19** (**1.14–1.24)**
Generalized anxiety disorder	699 (0.80)	16,437 (0.55)	**1.34** (**1.24–1.44)**	**1.30** (**1.20–1.40)**	473 (0.73)	596 (0.62)	**1.20** (**1.06–1.36)**
Agoraphobia	225 (0.26)	6135 (0.21)	1.13 (0.99–1.29)	1.09 (0.95–1.24)	144 (0.22)	213 (0.22)	1.02 (0.83–1.26)
Social anxiety disorder	635 (0.73)	16,886 (0.57)	**1.19** (**1.10–1.29)**	**1.15** (**1.06–1.24)**	457 (0.71)	583 (0.61)	**1.18** (**1.05–1.33)**
Anorexia nervosa	366 (0.42)	10,159 (0.34)	**1.32** (**1.19–1.46)**	**1.29** (**1.17–1.44)**	284 (0.44)	407 (0.43)	1.15 (0.98–1.33)
Substance use disorders	4456 (5.13)	108,145 (3.65)	**1.30** (**1.26–1.34)**	**1.26** (**1.23–1.31)**	3052 (4.74)	3885 (4.06)	**1.16** (**1.11–1.22)**
Any suicidal behavior^d^	3494 (4.02)	81,295 (2.74)	**1.37** (**1.32–1.42)**	**1.32** (**1.28–1.37)**	2443 (3.79)	3084 (3.22)	**1.19** (**1.13–1.25)**
Death by suicide	142 (0.16)	4248 (0.14)	0.94 (0.79–1.11)	0.92 (0.78–1.09)	99 (0.15)	116 (0.12)	1.18 (0.90–1.55)
Suicide attempt	3411 (3.92)	78,353 (2.64)	**1.40** (**1.35–1.45)**	**1.35** (**1.30–1.39)**	2385 (3.70)	2999 (3.13)	**1.20** (**1.13–1.26)**

Significant ORs are marked in bold

*OR* odds ratio, *CI* confidence interval

^a^Adjusted for individual’s year of birth and sex

^b^Additionally adjusted for county, maternal and paternal age at childbirth, parental highest educational level, parental lifetime history of psychiatric disorders, parental history of suicidal behavior, and number of siblings.

^c^Adjusted for year of birth and sex on both exposed and unexposed siblings

^d^Total numbers and percentage of the specific outcomes may not sum up to that of the combined outcomes as the study participants may have more than one specific outcome

### Additional analyses

#### Single versus joint exposure

**Fig. 1:**
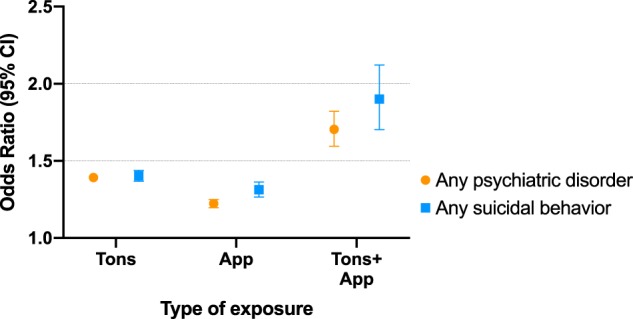
Association of the mutually exclusive categories of exposure, and for the joint exposure with any psychiatric disorder and with any suicidal behavior. *Note:* Individuals categorized into mutually exclusive categories, tonsillectomy only (Tons), acute appendicitis only (App), joint exposures (Tons + App), or none (reference). The association for any psychiatric disorder and any suicidal behavior was significantly higher among those exposed only to tonsillectomy (aOR = 1.39 [95% CI 1.37–1.41] for psychiatric disorders, aOR = 1.40 [95% CI 1.37–1.44] for suicidal behavior), as compared with only acute appendicitis (aOR = 1.22 [95% CI 1.20–1.25] for psychiatric disorders, aOR = 1.31 [95% CI 1.27–1.36] for suicidal behavior). The likelihood of the main outcomes was largest for those with both exposures (aOR = 1.70 [95% CI 1.59–1.82] for psychiatric disorders, aOR = 1.90 [95% CI 1.70–2.12] for suicidal behavior). The model adjusted for individual’s year of birth, sex, county, maternal and paternal age at childbirth, parental highest educational level, parental lifetime history of psychiatric disorders, parental history of suicidal behavior, and number of siblings

Figure 1 shows the results for mutually exclusive categories of single and joint exposure. The associations between exposure to only tonsillectomy and ‘any psychiatric disorder’ and ‘any suicidal behavior’ were significantly larger than the corresponding associations for exposure to only acute appendicitis. Individuals exposed to both tonsillectomy and acute appendicitis had significantly higher ORs than individuals with single exposures.

#### Incident cases

Analyses of incident cases revealed a significantly increased risk of subsequent development of ‘any psychiatric disorder’, ‘any suicidal behavior’, and the majority of the specific outcomes in individuals exposed to tonsillectomy in childhood (age 0–9 years) or in childhood and adolescence (age 0–19 years), with risks being higher in the latter group (Supplementary Table S5). The IRRs were slightly attenuated, but remained significant in the sibling analyses. Corresponding associations for exposure to acute appendicitis showed no significant results for the age of exposure 0–9 years, except for ADHD (Supplementary Table S6). Only few outcomes were significantly related to exposure to acute appendicitis at age 0–19 years, though most of associations decreased to non-significant in the analysis of discordant siblings.

#### Inpatient and outpatient records of psychiatric disorders and suicidal behavior

When excluding all individuals with an outcome recorded before 2001, the results were largely unchanged (Supplementary Table S7).

## Discussion

Our large population-based birth cohort study found that tonsillectomies, which in this study were employed as a proxy for chronic inflammation, are robustly associated with an increased likelihood of psychiatric disorders and suicidal behavior. This remained true in sibling analyses, which by definition adjust for unmeasured familial confounding. Moreover, the increased risk of developing psychiatric and suicidal outcomes was observed in the analyses of incident cases, suggesting that chronic inflammation may be implicated in the development of psychiatric disorders and suicidal behaviors. By contrast, acute appendicitis—a proxy for acute inflammation—was also associated with lifetime psychiatric disorders and suicidal behavior, but the magnitude of the associations was overall smaller than that seen for tonsillectomies. Furthermore, most associations observed in individuals with an exposure to acute appendicitis were no longer significant in sibling analyses and analysis of incident cases, suggesting that acute inflammatory processes may, in this context, play a less important role. Together, the results generally support the idea that inflammation of the MALT, particularly when chronic, plays an important role in psychiatric morbidity and suicidal behavior. The results are thus in line with the hypothesis that immune dysregulation is involved in the pathophysiology of psychiatric disorders and suicidal behavior^1,8^.

We also found a suggestive additive effect between tonsillectomies and appendicitis for both outcomes. By far, the highest ORs were obtained for individuals who had both tonsillectomy and appendicitis, compared with individuals who had either exposure alone. Future studies are needed to further explore the combined effect of chronic and acute inflammation on psychiatric and suicide outcomes but these results suggest a multiple-hit scenario^40^.

The pediatric autoimmune neuropsychiatric disorders associated with streptococcal infections (PANDAS) hypothesis claims a causal link between streptococcal tonsillitis and specific psychiatric disorders (OCD and TD) in a subgroup of vulnerable patients^41^. Our results do not support such specificity; instead, the association between proxies of inflammation and psychiatric disorders was broad and unspecific, highlighting the relevance of immunological dysfunction in psychiatric disorders in general. Similarly, in a Danish study, records of having performed a strep-A test (diagnostic test for streptococcal tonsillitis) were significantly associated with an increased risk of OCD and TD, but also other psychiatric disorders^42^. In fact, the risks for personality disorders and mood disorders were higher than the risks for OCD and TD in individuals with positive streptococcal tests (eTable 3 in ref. ^42^).

Our study has several strengths, including the population-based design, comprehensive collection of register-based data, a long follow-up, and the use of a sibling design which minimized the potential influence of familial confounding. Furthermore, our study has low risk of surveillance bias due to the exposures being severe conditions commonly requiring surgical interventions and, therefore, less likely to be influenced by help-seeking behavior.

A few limitations deserve discussion. First, our study is based on register-based proxies of chronic and acute inflammation within the MALT, rather than direct comparisons of different types of inflammation. The use of tonsillectomy and acute appendicitis as corresponding proxies implies the need to disentangle the impact of the inflammation per se from that of common interventions such as antibiotics^41^ or the surgical removal of immunological tissue because the vast majority of patients receive such treatments. For example, over the last 30 years in Sweden, 94.2% of children with acute appendicitis underwent appendectomy (in our cohort, 93.7% exposed to acute appendicitis underwent appendectomy) with the proportion of such operations being stable over time^19^. In addition, general anesthesia may be associated to an increased risk of psychiatric disorders^43^, perhaps due to toxic effects of the anesthetic drugs on the developing brain^44^. However, since both exposures in this study require surgery under general anesthesia, anesthesia alone is unlikely to explain that the magnitude of the association was significantly larger for tonsillectomy compared with appendicitis. Furthermore, a randomized trial did not find evidence for general anesthesia leading to differences in neurodevelopmental outcome compared with awake-regional anesthesia^45^. Second, since our data depends on the records of inpatient and outpatient visits, the date of the discharged diagnoses and operation may inaccurately represent the actual date of onset of inflammatory process and the outcome disorders. As such, we could not rule out the risk of reversed causation and, therefore, we mainly focused on exploring the association between the proxies of inflammation and outcomes without inferring causality. Third, despite a nationwide coverage, compulsory reporting, and high-validity of the NPR data for somatic and psychiatric conditions and surgery procedures^36^, the NPR does not include information from primary care and only keeps records from outpatient specialist care since 2001, leading to potential underestimation of less severe psychiatric cases. To account for such bias, we controlled for the year of birth and performed a sensitivity analysis where only outcomes recorded in 2001 or later were allowed. Finally, unavailability of data on indication for tonsillectomies precluded further analysis on the role of infections, hypertrophy, and sleeping apnea in the association between exposure to tonsillectomies and the outcomes of interest.

## Conclusions

Inflammation within the MALT, particularly chronic, is robustly associated with a broad range of psychiatric disorders and suicidal behavior, even after controlling for familial confounding. Additive effects from multiple insults appeared to strengthen the associations. Further research is needed to determine the underlying mechanisms of these associations.

## Supplementary information

Supplemental Material
