# Supplementary material for: Association of chronic and acute inflammation of the mucosa-associated lymphoid tissue with psychiatric disorders and suicidal behavior
Source: Transl Psychiatry. 2019 Sep 12;9:227. doi: 10.1038/s41398-019-0568-5 (PMC6742630; doi:10.1038/s41398-019-0568-5)
Supplement: Supplementary file 1 — Supplemental Material [file 41398_2019_568_MOESM1_ESM.docx]

# **Association of chronic and acute inflammation of the mucosa associated lymphoid tissue with psychiatric disorders and suicidal behavior**

Josef Isung, Kayoko Isomura, Catarina Almqvist, Paul Lichtenstein, Henrik Larsson, Tomas Wester, Christian Rück, Lorena Fernández de la Cruz, Anna Sidorchuk, and David Mataix-Cols

# **SUPPLEMENTAL MATERIAL**

# **Content:**

Supplementary information on ICD codes and operation codes used in the study.

Supplementary tables and figures referenced in the main manuscript.

**Table S1.** List of International Classification of Diseases (ICD) codes to collect records of tonsillectomy and acute appendicitis from the National Patient Register.

| **Conditions** | **ICD codes^a^** |
| --- | --- |
| Tonsillectomy | National operation codes 2710, 2720 from 1964-1996; and EMB10, EMB20, EMB30, and EMB99 from 1997-2013 |
| Acute appendicitis | Diagnoses coded 5400, 5409 (ICD-8) from 1969-1986; 5400, 5401, 5409 (ICD-9) from 1987-1996; K35 (ICD-10) from 1997-2013 |

^a^ In the National Patient Register the diagnoses are coded according to the Swedish version of ICD-codes in its 8^th^, 9^th^, or 10^th^ revision.

**Table S2.** List of Swedish International Classification of Diseases (ICD) codes to collect records of psychiatric disorders in the National Patient Register.

| **Diagnoses** | **ICD-8 codes^a^** | **ICD-9 codes^a^** | **ICD-10 codes^a^** | **Age constrains** |
| --- | --- | --- | --- | --- |
| Obsessive-compulsive disorder | 300.3 | 300D | F42 | If recorded at age 6 years or above |
| Tourette’s and chronic tic disorders | 306.2 following the algorithm described elsewhere.^1^ | 307C following the algorithm described elsewhere.^1^ | F95.1, F95.2 following the algorithm described elsewhere.^1^ | If recorded at age ≥3 years |
| Attention-deficit/hyperactivity disorder  Diagnosis defined through both ICD-code or prescription of ADHD drugs^b^ as recorded in the Prescribed Drug Register. The validity of this definition has been previously described.^2^ |  | 314 | F90  Prescribed Drug Register codes:^b^ N06BA01, N06BA02, N06BA04, N06BA09 | If recorded at age ≥3 years |
| Autism spectrum disorders |  | 299A | F84.0, F84.1, F84.8, F84.9 | If recorded at age ≥1 year |
| Schizophrenia and other psychotic disorders | 291, 295, 296.99, 297, 298, 299 | 291, 292, 295, 296X, 297, 298, 299 (except for 299A) | F10-F19 subsection .5, F20, F21, F22, F23, F25, F28, F29, F32.3 | If recorded at age ≥10 years |
| Bipolar disorder | 296, except 296.2 and 296.99 | 296, except 296B and 296X | F30, F31 | If recorded at age ≥10 years |
| Major depression disorder and other mood disorders | 296.2 | 296B | F32 (except for F32.3), F33, F34, F38, F39 | If recorded at age ≥10 years |
| Generalized anxiety disorder |  |  | F41.1 | If recorded at age ≥6 years |
| Agoraphobia |  |  | F40.0 | If recorded at age ≥6 years |
| Social anxiety disorder |  |  | F40.1 | If recorded at age ≥6 years |
| Anorexia nervosa |  | 307B | F50.0 | If recorded at age ≥8 years |
| Substance use disorders | 303, 304 | 303, 304, 305A, 305X | F10-F19 (except subsection .5) | If recorded at age ≥10 years |

^a^ In the National Patient Register the diagnoses are coded according to the Swedish version of ICD-codes in its 8^th^, 9^th^, or 10^th^ revision.

^b^ In the Prescribed Drug Register, drug classes are defined following the Anatomical Therapeutic Chemical (ATC) classification system codes.

**Table S3.** List of International Classification of Diseases (ICD) codes to define suicidal behavior in the National Patient Register^a^ and the Cause of Death Register.^b^

| **Diagnoses**^c^ | **ICD codes^a^** |
| --- | --- |
| Suicide and self-inflicted injury | E950-E959 (ICD-8 and ICD-9), if recorded at age ≥10 years |
| Intentional self-harm | X60-X84 (ICD-10), if recorded at age ≥10 years |
| Injury undetermined whether accidental or purposely inflicted | E980-E989 (ICD-8 and ICD-9), if recorded at age ≥10 years |
| Events of undetermined intent | Y10-Y34 (ICD-10), if recorded at age ≥10 years |

^a^ Diagnoses of suicide attempts were retrieved from the National Patient Register and were coded according to the Swedish version of ICD-codes in its 8^th^, 9^th^, or 10^th^ revision.

^b^ Diagnoses of death by suicide were retrieved from the Cause of Death Register using the same codes as for suicide attempts and were coded according to the International version of ICD-codes in its 8^th^, 9^th^, or 10^th^ revision.

^c^ Consistent with previous suicide research, both certain and undetermined causes were included in the definition of suicidal behavior.^3,4^

**Table S4.** Years between age of first record of an outcome in relation to age of exposure to tonsillectomy and to acute appendicitis for each individual psychiatric disorder and suicidal behavior.

|  | **Exposed to tonsillectomy** | | **Exposed to acute appendicitis** | |  |
| --- | --- | --- | --- | --- | --- |
|  | **Median** | **Interquartile range** | **Median** | **Interquartile range** |  |
| **Psychiatric Disorders** |  |  |  |  |  |
| Obsessive-compulsive disorder | 9 | 4–14 | 3 | 0–8 |  |
| Tourette’s and chronic tic disorders | 5 | 2–9 | 0 | -3–4 |  |
| Attention-deficit/hyperactivity disorder | 6 | 2–11 | 2 | 0–7 |  |
| Autism spectrum disorders | 6 | 2–10 | 0 | -2–5 |  |
| Schizophrenia and other psychotic disorders | 11 | 5–16 | 3 | -1–9 |  |
| Bipolar disorder | 12 | 6–16 | 5 | 0–9 |  |
| Major depression disorder and other mood disorders | 10 | 4–14 | 4 | 0–8 |  |
| Generalized anxiety disorder | 11 | 5–16 | 5 | 0–10 |  |
| Agoraphobia | 11,5 | 5–16 | 5 | 1–10 |  |
| Social anxiety disorder | 11 | 5–15 | 5 | 0–10 |  |
| Anorexia nervosa | 7 | 1–11 | 2 | -2–6 |  |
| Substance use disorders | 9 | 2–14 | 2 | -1–7 |  |
| **Suicidal Behavior** |  |  |  |  |  |
| Death by suicide | 13 | 7–17 | 8 | 4–12 |  |
| Suicide attempt | 8 | 2–13 | 2 | -1–7 |  |

*Note:* Negative values represent the situation when register-based records of psychiatric disorder and/or suicidal behavior preceded in time the record of tonsillectomy and acute appendicitis (i.e., age of outcome is lower than that of exposure), while positive values represent the opposite situation.

**Table S5.** Subsequent risk of developing psychiatric disorders and suicidal behavior in individuals exposed to tonsillectomy in childhood (age 0-9 years, upper panel) and in childhood and adolescence (age 0-19 years, lower panel) in the total cohort and in the full sibling analyses.

|  | **Total cohort** | | | |  | **Full siblings** | | |
| --- | --- | --- | --- | --- | --- | --- | --- | --- |
|  | **Exposed**  **No. (%)** | **Unexposed**  **No. (%)** | **Minimally adjusted IRR (95% CI)^a^** | **Fully adjusted**  **IRR (95% CI)^b^** |  | **Exposed**  **No. (%)** | **Unexposed**  **No. (%)** | **Minimally adjusted IRR (95% CI)^c^** |
| **Exposed at age between 0-9 years; followed up for outcome record from age 10 years** |  |  |  |  | |  |  |  |
| Total, no. | 119,174 | 2,822,665 |  |  | | 74,118 | 102,704 |  |
| Any psychiatric disorder^d^ | 13,363 (11.21) | 274,827 (9.74) | **1.30 (1.28-1.32)** | **1.25 (1.23-1.27)** | | 8,074 (10.89) | 10,001 (9.74) | **1.14 (1.11-1.17)** |
| Obsessive-compulsive disorder | 748 (0.63) | 17,033 (0.60) | **1.23 (1.15-1.33)** | **1.21 (1.13-1.31)** | | 450 (0.61) | 588 (0.57) | 1.12 (1.00-1.25) |
| Tourette’s and chronic tic disorders | 264 (0.22) | 3,420 (0.12) | **1.52 (1.34-1.73)** | **1.44 (1.26-1.63)** | | 154 (0.21) | 155 (0.15) | **1.31 (1.07-1.61)** |
| Attention-deficit/hyperactivity disorder | 5,319 (4.46) | 70,424 (2.49) | **1.56 (1.51-1.60)** | **1.45 (1.41-1.49)** | | 3,037 (4.10) | 3,119 (3.04) | **1.25 (1.20-1.31)** |
| Autism spectrum disorders | 896 (0.75) | 11,791 (0.42) | **1.55 (1.45-1.66)** | **1.49 (1.39-1.60)** | | 552 (0.74) | 476 (0.46) | **1.53 (1.37-1.71)** |
| Schizophrenia and other psychotic disorders | 593 (0.50) | 18,465 (0.65) | **1.15 (1.06-1.25)** | **1.11 (1.02-1.20)** | | 358 (0.48) | 436 (0.42) | **1.15 (1.01-1.30)** |
| Bipolar disorder | 717 (0.60) | 17,670 (0.63) | **1.41 (1.31-1.52)** | **1.35 (1.26-1.46)** | | 423 (0.57) | 560 (0.55) | **1.14 (1.02-1.28)** |
| Major depression disorder and other mood disorders | 5,162 (4.33) | 123,285 (4.37) | **1.29 (1.26-1.33)** | **1.24 (1.21-1.28)** | | 3,153 (4.25) | 4,325 (4.21) | **1.11 (1.07-1.15)** |
| Generalized anxiety disorder | 526 (0.44) | 15,526 (0.55) | **1.14 (1.05-1.25)** | **1.12 (1.03-1.22)** | | 330 (0.45) | 460 (0.45) | 1.04 (0.92-1.18) |
| Agoraphobia | 195 (0.16) | 5,815 (0.21) | **1.17 (1.01-1.35)** | 1.13 (0.97-1.30) | | 123 (0.17) | 160 (0.16) | 1.09 (0.88-1.34) |
| Social anxiety disorder | 610 (0.51) | 16,101 (0.57) | **1.14 (1.05-1.24)** | **1.11 (1.03-1.21)** | | 416 (0.56) | 499 (0.49) | **1.25 (1.11-1.40)** |
| Anorexia nervosa | 344 (0.29) | 9,748 (0.35) | 0.98 (0.88-1.09) | 1.00 (0.90-1.12) | | 229 (0.31) | 358 (0.35) | 1.01 (0.87-1.18) |
| Substance use disorders | 4,367 (3.66) | 102,094 (3.62) | **1.23 (1.20-1.27)** | **1.17 (1.13-1.20)** | | 2,630 (3.55) | 3,518 (3.43) | **1.07 (1.03-1.12)** |
| Any suicidal behavior^d^ | 129 (0.11) | 4,108 (0.15) | **1.29 (1.25-1.34)** | **1.23 (1.18-1.27)** | | 78 (0.11) | 98 (0.10) | **1.10 (1.04-1.16)** |
| Death by suicide | 3,188 (2.68) | 73,076 (2.59) | **1.23 (1.03-1.47)** | 1.13 (0.95-1.35) | | 1,949 (2.63) | 2,571 (2.50) | 1.10 (0.85-1.43) |
| Suicide attempt | 3,272 (2.75) | 75,930 (2.69) | **1.30 (1.25-1.35)** | **1.23 (1.19-1.28)** | | 2,002 (2.70) | 2,636 (2.57) | **1.10 (1.04-1.16)** |

**Table S5 (continued).** Subsequent risk of developing psychiatric disorders and suicidal behavior in individuals exposed to tonsillectomy in childhood (age 0-9 years, upper panel) and in childhood and adolescence (age 0-19 years, lower panel) in the total cohort and in the full sibling analyses.

|  | **Total cohort** | | | |  | **Full siblings** | | |
| --- | --- | --- | --- | --- | --- | --- | --- | --- |
|  | **Exposed**  **No. (%)** | **Unexposed**  **No. (%)** | **Minimally adjusted IRR (95% CI)^a^** | **Fully adjusted IRR (95% CI)^b^** |  | **Exposed**  **No. (%)** | **Unexposed**  **No. (%)** | **Minimally adjusted IRR (95% CI)^c^** |
| **Exposed at age between 0-19 years; followed up for outcome record from age 20 years** |  |  |  |  | |  |  |  |
| Total, no. | 168,096 | 2,664,333 |  |  | | 108,431 | 144,218 |  |
| Any psychiatric disorder^d^ | 8,850 (5.26) | 136,146 (5.11) | **1.36 (1.33-1.39)** | **1.30 (1.28-1.33)** | | 5,439 (5.02) | 6,360 (4.41) | **1.19 (1.15-1.23)** |
| Obsessive-compulsive disorder | 486 (0.29) | 8,961 (0.34) | **1.12 (1.03-1.23)** | **1.11 (1.01-1.21)** | | 276 (0.25) | 373 (0.26) | 0.96 (0.83-1.11) |
| Tourette’s and chronic tic disorders | 50 (0.03) | 664 (0.02) | **1.67 (1.24-2.25)** | **1.63 (1.22-2.19)** | | 29 (0.03) | 29 (0.02) | 1.53 (0.93-2.53) |
| Attention-deficit/hyperactivity disorder | 1,667 (0.99) | 21,416 (0.80) | **1.62 (1.54-1.70)** | **1.52 (1.45-1.60)** | | 980 (0.90) | 1,046 (0.73) | **1.27 (1.17-1.38)** |
| Autism spectrum disorders | 158 (0.09) | 2,867 (0.11) | 1.14 (0.97-1.34) | 1.14 (0.97-1.34) | | 101 (0.09) | 131 (0.09) | 1.07 (0.84-1.37) |
| Schizophrenia and other psychotic disorders | 537 (0.32) | 11,160 (0.42) | **1.11 (1.02-1.22)** | 1.08 (0.99-1.18) | | 336 (0.31) | 412 (0.29) | **1.18 (1.03-1.35)** |
| Bipolar disorder | 807 (0.48) | 11,325 (0.43) | **1.52 (1.41-1.63)** | **1.46 (1.36-1.57)** | | 509 (0.47) | 527 (0.37) | **1.27 (1.14-1.43)** |
| Major depression disorder and other mood disorders | 4,863 (2.89) | 74,358 (2.79) | **1.38 (1.34-1.42)** | **1.33 (1.29-1.37)** | | 3,001 (2.77) | 3,446 (2.39) | **1.19 (1.14-1.25)** |
| Generalized anxiety disorder | 640 (0.38) | 10,323 (0.39) | **1.32 (1.22-1.44)** | **1.30 (1.20-1.40)** | | 394 (0.36) | 448 (0.31) | **1.18 (1.04-1.33)** |
| Agoraphobia | 241 (0.14) | 4,054 (0.15) | **1.27 (1.12-1.45)** | **1.22 (1.07-1.39)** | | 150 (0.14) | 158 (0.11) | 1.23 (1.00-1.51) |
| Social anxiety disorder | 537 (0.32) | 9,906 (0.37) | **1.13 (1.04-1.23)** | **1.10 (1.01-1.20)** | | 347 (0.32) | 392 (0.27) | **1.16 (1.02-1.33)** |
| Anorexia nervosa | 124 (0.07) | 2,198 (0.08) | 1.06 (0.88-1.27) | 1.06 (0.88-1.27) | | 82 (0.08) | 99 (0.07) | 1.14 (0.86-1.50) |
| Substance use disorders | 3,404 (2.03) | 48,641 (1.83) | **1.48 (1.43-1.54)** | **1.39 (1.34-1.44)** | | 2,027 (1.87) | 2,342 (1.62) | **1.26 (1.19-1.33)** |
| Any suicidal behavior^d^ | 129 (0.08) | 2,490 (0.09) | **1.46 (1.40-1.53)** | **1.38 (1.32-1.44)** | | 86 (0.08) | 92 (0.06) | **1.20 (1.13-1.29)** |
| Death by suicide | 2,175 (1.29) | 32,130 (1.21) | **1.31 (1.10-1.56)** | **1.21 (1.01-1.44)** | | 1,312 (1.21) | 1,561 (1.08) | **1.47 (1.11-1.94)** |
| Suicide attempt | 2,267 (1.35) | 33,990 (1.28) | **1.47 (1.41-1.54)** | **1.39 (1.33-1.45)** | | 1,374 (1.27) | 1,628 (1.13) | **1.20 (1.12-1.28)** |

*Note:* Individuals exposed to tonsillectomy from age 0 to 9 years were compared to individuals free from exposure at any age, and were followed from age 10 years for the incident cases of psychiatric disorders and suicidal behavior. Exposed and unexposed participants were excluded from the analysis if received an outcome record before age 10 years. The same analysis was repeated for age of exposure from age 0 to 19 years compared to individuals free from exposure at any age, with follow-up for an outcome record starting from age 20 years. From the second analysis, all individuals with an outcome record before age 20 years were excluded.

^a^ Adjusted for individual’s year of birth and sex

^b^ Additionally adjusted for county, maternal and paternal age at childbirth, parental highest educational level, parental lifetime history of psychiatric disorders, parental history of suicidal behavior, and number of siblings

^c^ Adjusted for year of birth and sex on both exposed and unexposed siblings

^d^ Total numbers and percentage of the specific outcomes may not sum up to that of the combined outcomes as the study participants may have more than one specific outcome

Abbreviation: IRR, incidence rate ratio; CI, confidence intervals

Significant ORs are marked in bold

**Table S6.** Subsequent risk of developing psychiatric disorders and suicidal behavior in individuals exposed to acute appendicitis in childhood (age 0-9 years, upper panel) and in childhood and adolescence (age 0-19 years, lower panel) in the total cohort and in the full sibling analyses.

|  | **Total cohort** | | | |  | **Full siblings** | | |
| --- | --- | --- | --- | --- | --- | --- | --- | --- |
|  | **Exposed**  **No. (%)** | **Unexposed**  **No. (%)** | **Min-adjusted IRR (95% CI)^a^** | **Fully adjusted IRR (95% CI)^b^** |  | **Exposed**  **No. (%)** | **Unexposed**  **No. (%)** | **Min-adjusted IRR (95% CI)^c^** |
| **Exposed at age between 0-9 years; followed up for outcome record from age 10 years** |  |  |  |  | |  |  |  |
| Total, no. | 13,695 | 2,944,076 |  |  | | 9,610 | 13,904 |  |
| Any psychiatric disorder^d^ | 1,390 (10.15) | 290,678 (9.87) | 1.05 (1.00-1.10) | 1.03 (0.98-1.09) | | 941 (9.79) | 1,273 (9.16) | 0.94 (0.88-1.00) |
| Obsessive-compulsive disorder | 74 (0.54) | 17,883 (0.61) | 0.92 (0.73-1.16) | 0.91 (0.72-1.14) | | 49 (0.51) | 82 (0.59) | 0.82 (0.62-1.10) |
| Tourette’s and chronic tic disorders | 23 (0.17) | 3,687 (0.13) | 1.23 (0.82-1.85) | 1.21 (0.80-1.82) | | 16 (0.17) | 21 (0.15) | 1.27 (0.74-2.16) |
| Attention-deficit/hyperactivity disorder | 461 (3.37) | 76,395 (2.59) | **1.23 (1.13-1.35)** | **1.21 (1.11-1.32)** | | 312 (3.25) | 352 (2.53) | 1.08 (0.96-1.22) |
| Autism spectrum disorders | 62 (0.45) | 12,637 (0.43) | 1.00 (0.78-1.28) | 0.98 (0.77-1.26) | | 45 (0.47) | 70 (0.50) | 0.94 (0.70-1.28) |
| Schizophrenia and other psychotic disorders | 86 (0.63) | 19,051 (0.65) | 0.96 (0.78-1.19) | 0.95 (0.77-1.17) | | 55 (0.57) | 68 (0.49) | 1.00 (0.76-1.31) |
| Bipolar disorder | 73 (0.53) | 18,687 (0.63) | 0.88 (0.70-1.10) | 0.86 (0.68-1.08) | | 53 (0.55) | 86 (0.62) | 0.87 (0.66-1.15) |
| Major depression disorder and other mood disorders | 581 (4.24) | 130,081 (4.42) | 1.00 (0.93-1.09) | 0.99 (0.91-1.07) | | 397 (4.13) | 600 (4.32) | **0.89 (0.81-0.98)** |
| Generalized anxiety disorder | 76 (0.55) | 16,222 (0.55) | 1.05 (0.84-1.32) | 1.03 (0.82-1.29) | | 47 (0.49) | 70 (0.50) | 0.91 (0.68-1.23) |
| Agoraphobia | 26 (0.19) | 6,111 (0.21) | 0.95 (0.64-1.39) | 0.92 (0.62-1.35) | | 17 (0.18) | 19 (0.14) | 0.92 (0.56-1.51) |
| Social anxiety disorder | 82 (0.60) | 16,818 (0.57) | 1.08 (0.87-1.34) | 1.06 (0.86-1.32) | | 58 (0.60) | 70 (0.50) | 1.09 (0.83-1.43) |
| Anorexia nervosa | 42 (0.31) | 10,089 (0.34) | 0.99 (0.73-1.33) | 0.98 (0.72-1.32) | | 34 (0.35) | 58 (0.42) | 0.94 (0.66-1.33) |
| Substance use disorders | 472 (3.45) | 107,677 (3.66) | 0.95 (0.87-1.04) | 0.94 (0.86-1.02) | | 318 (3.31) | 422 (3.04) | **0.86 (0.77-0.97)** |
| Any suicidal behavior^d^ | 26 (0.19) | 4,239 (0.14) | 1.09 (0.99-1.20) | 1.07 (0.97-1.18) | | 14 (0.15) | 13 (0.09) | 0.94 (0.83-1.07) |
| Death by suicide | 373 (2.72) | 77,236 (2.62) | 1.26 (0.86-1.85) | 1.23 (0.84-1.81) | | 253 (2.63) | 358 (2.57) | 1.32 (0.75-2.29) |
| Suicide attempt | 396 (2.89) | 80,173 (2.72) | 1.07 (0.97-1.18) | 1.05 (0.95-1.16) | | 266 (2.77) | 366 (2.63) | 0.92 (0.81-1.05) |

**Table S6 (continued).** Subsequent risk of developing psychiatric disorders and suicidal behavior in individuals exposed to acute appendicitis in childhood (age 0-9 years, upper panel) and in childhood and adolescence (age 0-19 years, lower panel) in the total cohort and in the full sibling analyses.

|  | **Total cohort** | | | |  | **Full siblings** | | |
| --- | --- | --- | --- | --- | --- | --- | --- | --- |
|  | **Exposed**  **No. (%)** | **Unexposed**  **No. (%)** | **Minimally adjusted IRR (95% CI)^a^** | **Fully adjusted IRR (95% CI)^b^** |  | **Exposed**  **No. (%)** | **Unexposed**  **No. (%)** | **Minimally adjusted IRR (95% CI)^c^** |
| **Exposed at age between 0-19 years; followed up for outcome record from age 20 years** |  |  |  |  | |  |  |  |
| Total, no. | 49,485 | 2,774,360 |  |  | | 37,902 | 54,184 |  |
| Any psychiatric disorder^d^ | 2,673 (5.40) | 141,781 (5.11) | **1.20 (1.15-1.24)** | **1.17 (1.13-1.22)** | | 1,861 (4.91) | 2,551 (4.71) | 1.05 (1.00-1.11) |
| Obsessive-compulsive disorder | 149 (0.30) | 9,264 (0.33) | 1.04 (0.89-1.23) | 1.02 (0.87-1.20) | | 108 (0.28) | 168 (0.31) | 0.90 (0.73-1.12) |
| Tourette’s and chronic tic disorders | 16 (0.03) | 696 (0.03) | 1.42 (0.87-2.33) | 1.38 (0.84-2.27) | | 11 (0.03) | 9 (0.02) | 1.32 (0.65-2.70) |
| Attention-deficit/hyperactivity disorder | 453 (0.92) | 22,535 (0.81) | **1.24 (1.13-1.36)** | **1.22 (1.11-1.33)** | | 299 (0.79) | 380 (0.70) | 1.07 (0.94-1.23) |
| Autism spectrum disorders | 37 (0.07) | 2,953 (0.11) | 0.76 (0.55-1.06) | 0.75 (0.54-1.03) | | 23 (0.06) | 69 (0.13) | **0.56 (0.36-0.88)** |
| Schizophrenia and other psychotic disorders | 173 (0.35) | 11,410 (0.41) | 0.97 (0.84-1.13) | 0.96 (0.83-1.11) | | 115 (0.30) | 163 (0.30) | 1.01 (0.82-1.25) |
| Bipolar disorder | 208 (0.42) | 11,863 (0.43) | **1.17 (1.02-1.34)** | 1.13 (0.99-1.30) | | 151 (0.40) | 200 (0.37) | 1.08 (0.90-1.30) |
| Major depression disorder and other mood disorders | 1,405 (2.84) | 77,506 (2.79) | **1.19 (1.13-1.25)** | **1.16 (1.10-1.22)** | | 970 (2.56) | 1,424 (2.63) | 1.02 (0.95-1.09) |
| Generalized anxiety disorder | 197 (0.40) | 10,705 (0.39) | **1.23 (1.06-1.41)** | **1.20 (1.04-1.38)** | | 124 (0.33) | 193 (0.36) | 0.96 (0.79-1.18) |
| Agoraphobia | 68 (0.14) | 4,218 (0.15) | 1.06 (0.83-1.34) | 1.03 (0.81-1.31) | | 46 (0.12) | 66 (0.12) | 0.98 (0.71-1.36) |
| Social anxiety disorder | 192 (0.39) | 10,221 (0.37) | **1.19 (1.03-1.37)** | 1.16 (1.00-1.33) | | 139 (0.37) | 172 (0.32) | 1.14 (0.94-1.39) |
| Anorexia nervosa | 56 (0.11) | 2,245 (0.08) | **1.70 (1.30-2.21)** | **1.66 (1.28-2.17)** | | 45 (0.12) | 63 (0.12) | 1.29 (0.91-1.82) |
| Substance use disorders | 1,016 (2.05) | 50,856 (1.83) | **1.23 (1.15-1.30)** | **1.20 (1.13-1.28)** | | 696 (1.84) | 867 (1.60) | **1.10 (1.01-1.20)** |
| Any suicidal behavior^d^ | 51 (0.10) | 2,546 (0.09) | **1.27 (1.18-1.37)** | **1.23 (1.14-1.32)** | | 36 (0.09) | 29 (0.05) | 1.07 (0.96-1.19) |
| Death by suicide | 668 (1.35) | 33,504 (1.21) | 1.29 (0.98-1.70) | 1.26 (0.96-1.67) | | 462 (1.22) | 640 (1.18) | **1.72 (1.13-2.62)** |
| Suicide attempt | 705 (1.42) | 35,414 (1.28) | **1.27 (1.18-1.37)** | **1.23 (1.14-1.32)** | | 487 (1.28) | 662 (1.22) | 1.05 (0.95-1.17) |

*Note:* Individuals exposed to acute appendicitis from age 0 to 9 years were compared to individuals free from exposure at any age, and were followed from age 10 years for the incident cases of psychiatric disorders and suicidal behavior. Exposed and unexposed participants were excluded from the analysis if received an outcome record before age 10 years. The same analysis was repeated for age of exposure from age 0 to 19 years compared to individuals free from exposure at any age, with follow-up for an outcome record starting from age 20 years. From the second analysis, all individuals with an outcome record before age 20 years were excluded.

^a^ Adjusted for individual’s year of birth and sex

^b^ Additionally adjusted for county, maternal and paternal age at childbirth, parental highest educational level, parental lifetime history of psychiatric disorders, parental history of suicidal behavior, and number of siblings

^c^ Adjusted for year of birth and sex on both exposed and unexposed siblings

^d^ Total numbers and percentage of the specific outcomes may not sum up to that of the combined outcomes as the study participants may have more than one specific outcome

Abbreviation: IRR, incidence rate ratio; CI, confidence intervals

Significant ORs are marked in bold

**Table S7.** Association of exposure to tonsillectomy (upper panel) and to acute appendicitis (lower panel) with psychiatric disorders and suicidal behavior after excluding individuals with a record of outcome before 2001 in the total cohort and in the full sibling analyses.

|  | **Total cohort** | | | |  | **Full siblings** | | |
| --- | --- | --- | --- | --- | --- | --- | --- | --- |
|  | **Exposed**  **No. (%)** | **Unexposed**  **No. (%)** | **Minimally adjusted OR (95% CI)^a^** | **Fully adjusted OR (95% CI)^b^** |  | **Exposed**  **No. (%)** | **Unexposed**  **No. (%)** | **Minimally adjusted OR (95% CI)^c^** |
| **Exposed to tonsillectomy** |  |  |  |  | |  |  |  |
| Total, no. | 207 928 | 2 808 939 |  |  | | 133 965 | 179 833 |  |
| Any psychiatric disorder^d^ | 27 463 (13.21) | 267 355 (9.52) | **1.46 (1.44-1.48)** | **1.40 (1.38-1.42)** | | 16 776 (12.52) | 18 845 (10.48) | **1.22 (1.20-1.25)** |
| Obsessive-compulsive disorder | 1 443 (0.69) | 16 380 (0.58) | **1.22 (1.15-1.29)** | **1.19 (1.13-1.26)** | | 875 (0.65) | 1 097 (0.61) | 1.07 (0.98-1.16) |
| Tourette’s and chronic tic disorders | 568 (0.27) | 4 562 (0.16) | **1.53 (1.40-1.67)** | **1.42 (1.30-1.55)** | | 314 (0.23) | 329 (0.18) | **1.26 (1.08-1.47)** |
| Attention-deficit/hyperactivity disorder | 10 397 (5.00) | 81 015 (2.88) | **1.66 (1.62-1.69)** | **1.54 (1.51-1.57)** | | 5 854 (4.37) | 6 091 (3.39) | **1.29 (1.25-1.34)** |
| Autism spectrum disorders | 1 883 (0.91) | 16 042 (0.57) | **1.45 (1.38-1.52)** | **1.39 (1.32-1.46)** | | 1 079 (0.81) | 1 102 (0.61) | **1.29 (1.19-1.40)** |
| Schizophrenia and other psychotic disorders | 1 047 (0.50) | 14 322 (0.51) | **1.11 (1.04-1.18)** | 1.07 (1.00-1.14) | | 661 (0.49) | 861 (0.48) | 1.07 (0.97-1.18) |
| Bipolar disorder | 1 687 (0.81) | 15 828 (0.56) | **1.56 (1.48-1.64)** | **1.48 (1.40-1.55)** | | 1 042 (0.78) | 1 154 (0.64) | **1.20 (1.11-1.31)** |
| Major depression disorder and other mood disorders | 11 571 (5.56) | 115 909 (4.13) | **1.46 (1.43-1.49)** | **1.39 (1.37-1.42)** | | 7 308 (5.46) | 8 117 (4.51) | **1.22 (1.18-1.26)** |
| Generalized anxiety disorder | 1 286 (0.62) | 14 559 (0.52) | **1.29 (1.22-1.37)** | **1.26 (1.19-1.33)** | | 813 (0.61) | 964 (0.54) | **1.13 (1.03-1.24)** |
| Agoraphobia | 472 (0.23) | 5 435 (0.19) | **1.30 (1.18-1.43)** | **1.24 (1.13-1.36)** | | 299 (0.22) | 350 (0.19) | 1.15 (0.99-1.35) |
| Social anxiety disorder | 1 258 (0.61) | 15 220 (0.54) | **1.19 (1.12-1.26)** | **1.15 (1.09-1.22)** | | 841 (0.63) | 959 (0.53) | **1.18 (1.08-1.30)** |
| Anorexia nervosa | 593 (0.29) | 7 938 (0.28) | 0.97 (0.89-1.05) | 0.99 (0.91-1.07) | | 402 (0.30) | 562 (0.31) | 0.93 (0.82-1.06) |
| Substance use disorders | 8 661 (4.17) | 85 621 (3.05) | **1.46 (1.42-1.49)** | **1.38 (1.35-1.41)** | | 5 444 (4.06) | 6 161 (3.43) | **1.21 (1.17-1.26)** |
| Any suicidal behavior^d^ | 6 538 (3.14) | 63 001 (2.24) | **1.47 (1.44-1.51)** | **1.39 (1.35-1.43)** | | 4 213 (3.14) | 4 692 (2.61) | **1.22 (1.17-1.27)** |
| Death by suicide | 203 (0.10) | 2 801 (0.10) | 1.16 (1.00-1.33) | 1.07 (0.92-1.23) | | 135 (0.10) | 169 (0.09) | 1.15 (0.92-1.45) |
| Suicide attempt | 6 404 (3.08) | 61 001 (2.17) | **1.49 (1.45-1.53)** | **1.40 (1.37-1.44)** | | 4 122 (3.08) | 4 574 (2.54) | **1.22 (1.17-1.27)** |

**Table S7 (continued).** Association of exposure to tonsillectomy (upper panel) and to acute appendicitis (lower panel) with psychiatric disorders and suicidal behavior after excluding individuals with a record of outcome before 2001in the total cohort and in the full sibling analyses.

|  | **Total cohort** | | | |  | **Full siblings** | | |
| --- | --- | --- | --- | --- | --- | --- | --- | --- |
|  | **Exposed**  **No. (%)** | **Unexposed**  **No. (%)** | **Minimally adjusted**  **OR (95% CI)^a^** | **Fully adjusted OR (95% CI)^b^** |  | **Exposed**  **No. (%)** | **Unexposed**  **No. (%)** | **Minimally adjusted OR (95% CI)^c^** |
| **Exposed to acute appendicitis** |  |  |  |  | |  |  |  |
| Total, no. | 85 636 | 2 931 231 |  |  | | 63 612 | 94 552 |  |
| Any psychiatric disorder^d^ | 10 407 (12.15) | 284 411 (9.70) | **1.27 (1.25-1.30)** | **1.25 (1.22-1.28)** | | 7 364 (11.58) | 9 826 (10.39) | **1.14 (1.11-1.18)** |
| Obsessive-compulsive disorder | 579 (0.68) | 17 244 (0.59) | **1.13 (1.03-1.22)** | **1.11 (1.02-1.20)** | | 403 (0.63) | 618 (0.65) | 0.99 (0.87-1.12) |
| Tourette’s and chronic tic disorders | 146 (0.17) | 4 984 (0.17) | **1.19 (1.01-1.40)** | 1.17 (0.99-1.38) | | 104 (0.16) | 114 (0.12) | **1.32 (1.01-1.73)** |
| Attention-deficit/hyperactivity disorder | 2 851 (3.33) | 88 561 (3.02) | **1.27 (1.22-1.32)** | **1.24 (1.19-1.29)** | | 1 956 (3.07) | 2 586 (2.74) | **1.14 (1.08-1.21)** |
| Autism spectrum disorders | 413 (0.48) | 17 512 (0.60) | 0.98 (0.89-1.09) | 0.96 (0.87-1.06) | | 294 (0.46) | 485 (0.51) | 0.91 (0.79-1.06) |
| Schizophrenia and other psychotic disorders | 550 (0.64) | 14 819 (0.51) | **1.12 (1.03-1.22)** | **1.09 (1.01-1.19)** | | 373 (0.59) | 503 (0.53) | 1.08 (0.94-1.23) |
| Bipolar disorder | 740 (0.86) | 16 775 (0.57) | **1.40 (1.30-1.51)** | **1.36 (1.26-1.46)** | | 514 (0.81) | 634 (0.67) | **1.24 (1.10-1.39)** |
| Major depression disorder and other mood disorders | 5 098 (5.95) | 122 382 (4.18) | **1.37 (1.33-1.41)** | **1.34 (1.30-1.38)** | | 3 607 (5.67) | 4 658 (4.93) | **1.19 (1.14-1.25)** |
| Generalized anxiety disorder | 647 (0.76) | 15 198 (0.52) | **1.35 (1.25-1.47)** | **1.31 (1.21-1.42)** | | 441 (0.69) | 565 (0.60) | **1.19 (1.05-1.34)** |
| Agoraphobia | 209 (0.24) | 5 698 (0.19) | 1.14 (0.99-1.31) | 1.10 (0.96-1.26) | | 136 (0.21) | 203 (0.21) | 1.01 (0.82-1.26) |
| Social anxiety disorder | 591 (0.69) | 15 887 (0.54) | **1.19 (1.09-1.29)** | **1.15 (1.06-1.25)** | | 430 (0.68) | 564 (0.60) | **1.15 (1.02-1.30)** |
| Anorexia nervosa | 300 (0.35) | 8 231 (0.28) | **1.44 (1.28-1.61)** | **1.41 (1.26-1.59)** | | 239 (0.38) | 337 (0.36) | 1.19 (1.00-1.40) |
| Substance use disorders | 3 756 (4.39) | 90 526 (3.09) | **1.35 (1.30-1.39)** | **1.31 (1.27-1.36)** | | 2 641 (4.15) | 3 308 (3.50) | **1.19 (1.13-1.25)** |
| Any suicidal behavior^d^ | 2 894 (3.38) | 66 645 (2.27) | **1.43 (1.38-1.49)** | **1.38 (1.32-1.43)** | | 2 102 (3.30) | 2 619 (2.77) | **1.21 (1.14-1.28)** |
| Death by suicide | 107 (0.12) | 2 897 (0.10) | 1.07 (0.88-1.30) | 1.05 (0.86-1.27) | | 82 (0.13) | 81 (0.09) | **1.41 (1.03-1.92)** |
| Suicide attempt | 2 830 (3.30) | 64 575 (2.20) | **1.45 (1.40-1.51)** | **1.39 (1.34-1.45)** | | 2 053 (3.23) | 2 560 (2.71) | **1.21 (1.14-1.28)** |

^a^ Adjusted for individual’s year of birth and sex

^b^ Additionally adjusted for county, maternal and paternal age at childbirth, parental highest educational level, parental lifetime history of psychiatric disorders, parental history of suicidal behavior, and number of siblings

^c^ Adjusted for year of birth and sex on both exposed and unexposed siblings

^d^ Total numbers and percentage of the specific outcomes may not sum up to that of the combined outcomes as the study participants may have more than one specific outcome

Abbreviation: OR, odds ratio; CI, confidence intervals

Significant ORs are marked in bold

**Figure S1.** Years (in 5-year intervals) between age of first record of an outcome in relation to the age of exposure to tonsillectomy for each individual psychiatric disorder and suicidal behavior.


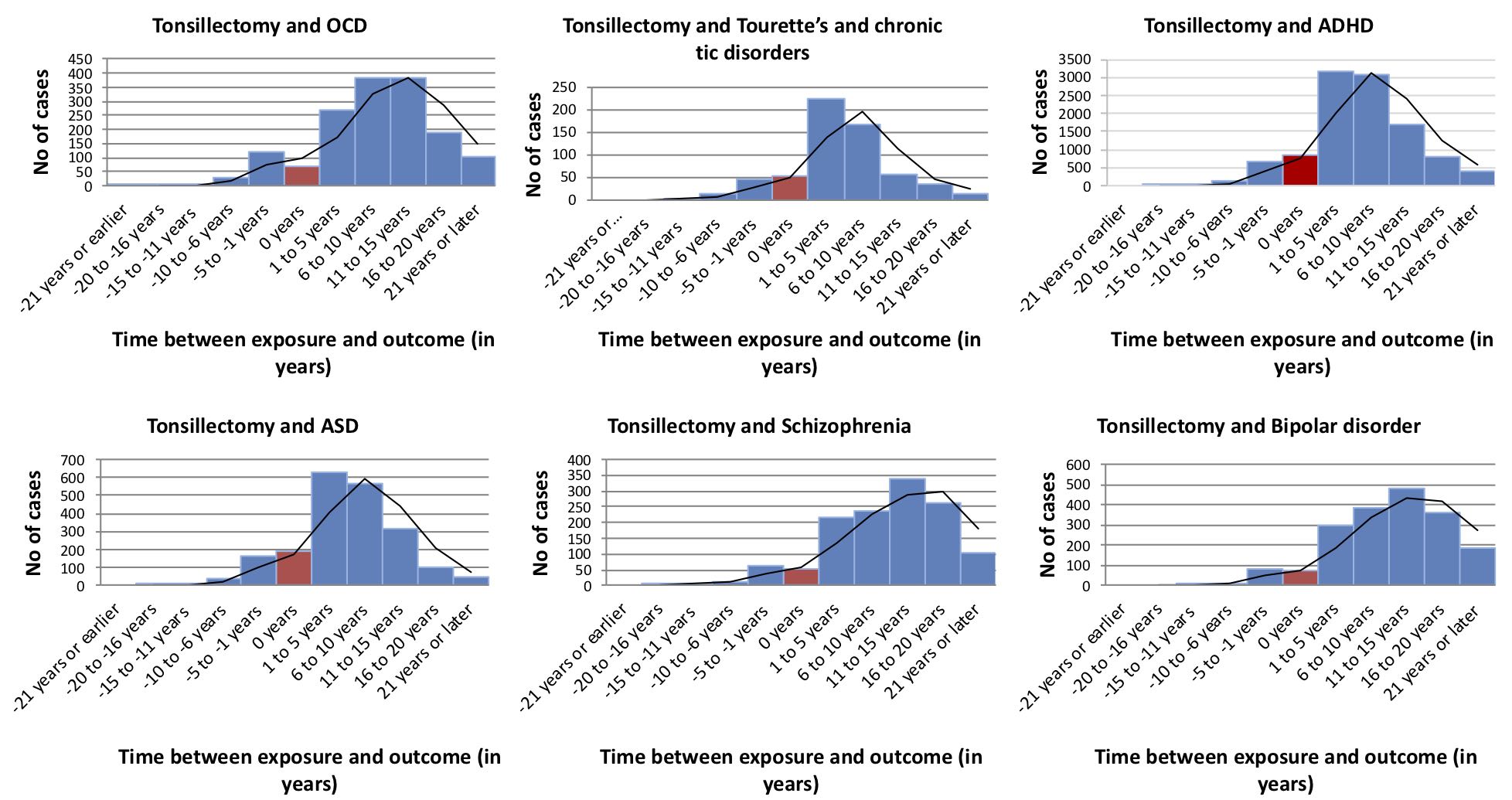


*Note:* Negative values represent the situation when register-based records of psychiatric disorder and/or suicidal behavior preceded in time the record of tonsillectomy and acute appendicitis (i.e., age of outcome is lower than that of exposure), while positive values represent the opposite situation. A value ‘zero’ represents individuals for whom the age at exposure was equal to age of outcome (presented by red bar to ease visualization). A trend line represents a moving average.

Abbreviation: OCD, Obsessive-compulsive disorder; ADHD, Attention-deficit/hyperactivity disorder; ASD, Autism spectrum disorder

**Figure S1 (continued).** Years (in 5-year intervals) between age of first record of an outcome in relation to the age of exposure to tonsillectomy for each individual psychiatric disorder and suicidal behavior.


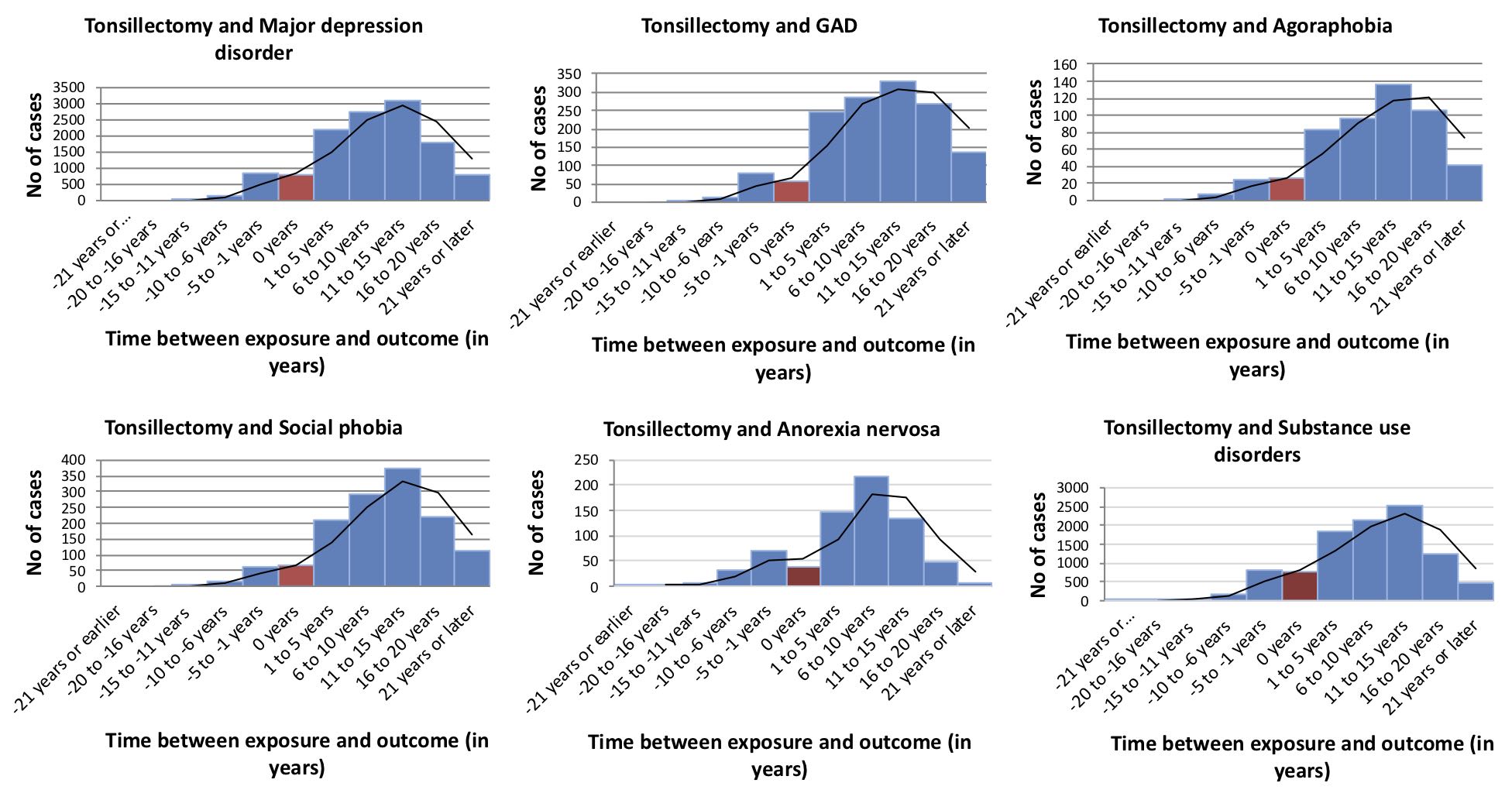


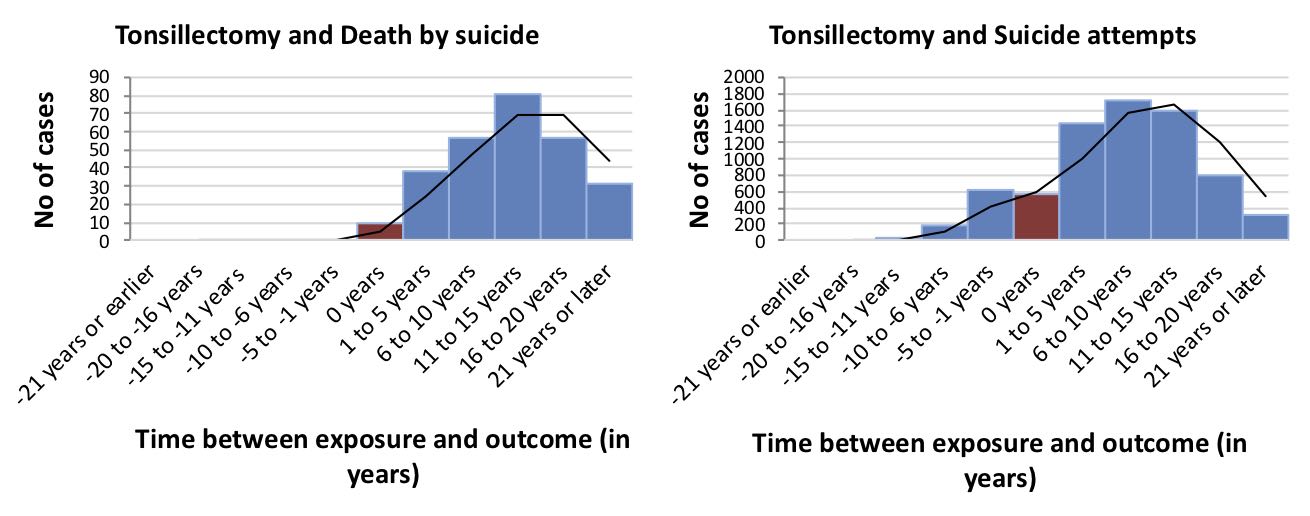


*Note:* Negative values represent the situation when register-based records of psychiatric disorder and/or suicidal behavior preceded in time the record of tonsillectomy and acute appendicitis (i.e., age of outcome is lower than that of exposure), while positive values represent the opposite situation. A value ‘zero’ represents individuals for whom the age at exposure was equal to age of outcome (presented by red bar to ease visualization). A trend line represents a moving average.

Abbreviation: GAD, Generalized anxiety disorder

**Figure S2.** Years (in 5-year intervals) between age of first record of an outcome in relation to the age of exposure to acute appendicitis for each individual psychiatric disorder and suicidal behavior.


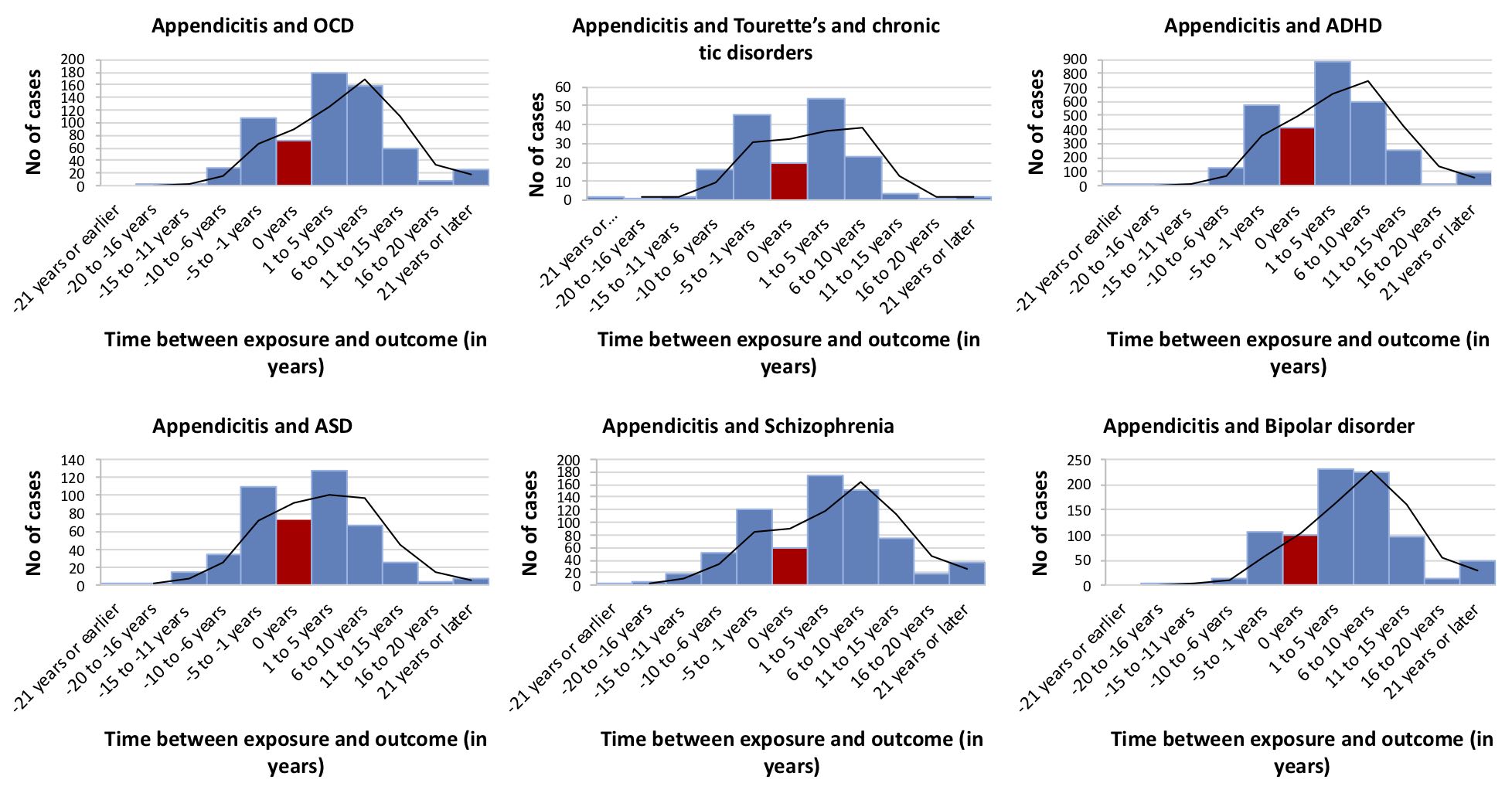


*Note:* Negative values represent the situation when register-based records of psychiatric disorder and/or suicidal behavior preceded in time the record of tonsillectomy and acute appendicitis (i.e., age of outcome is lower than that of exposure), while positive values represent the opposite situation. A value ‘zero’ represents individuals for whom the age at exposure was equal to age of outcome (presented by red bar to ease visualization). A trend line represents a moving average.

Abbreviation: OCD, Obsessive-compulsive disorder; ADHD, Attention-deficit/hyperactivity disorder; ASD, Autism spectrum disorder

**Figure S2 (continued).** Years (in 5-year intervals) between age of first record of an outcome in relation to the age of exposure to acute appendicitis for each individual psychiatric disorder and suicidal behavior.


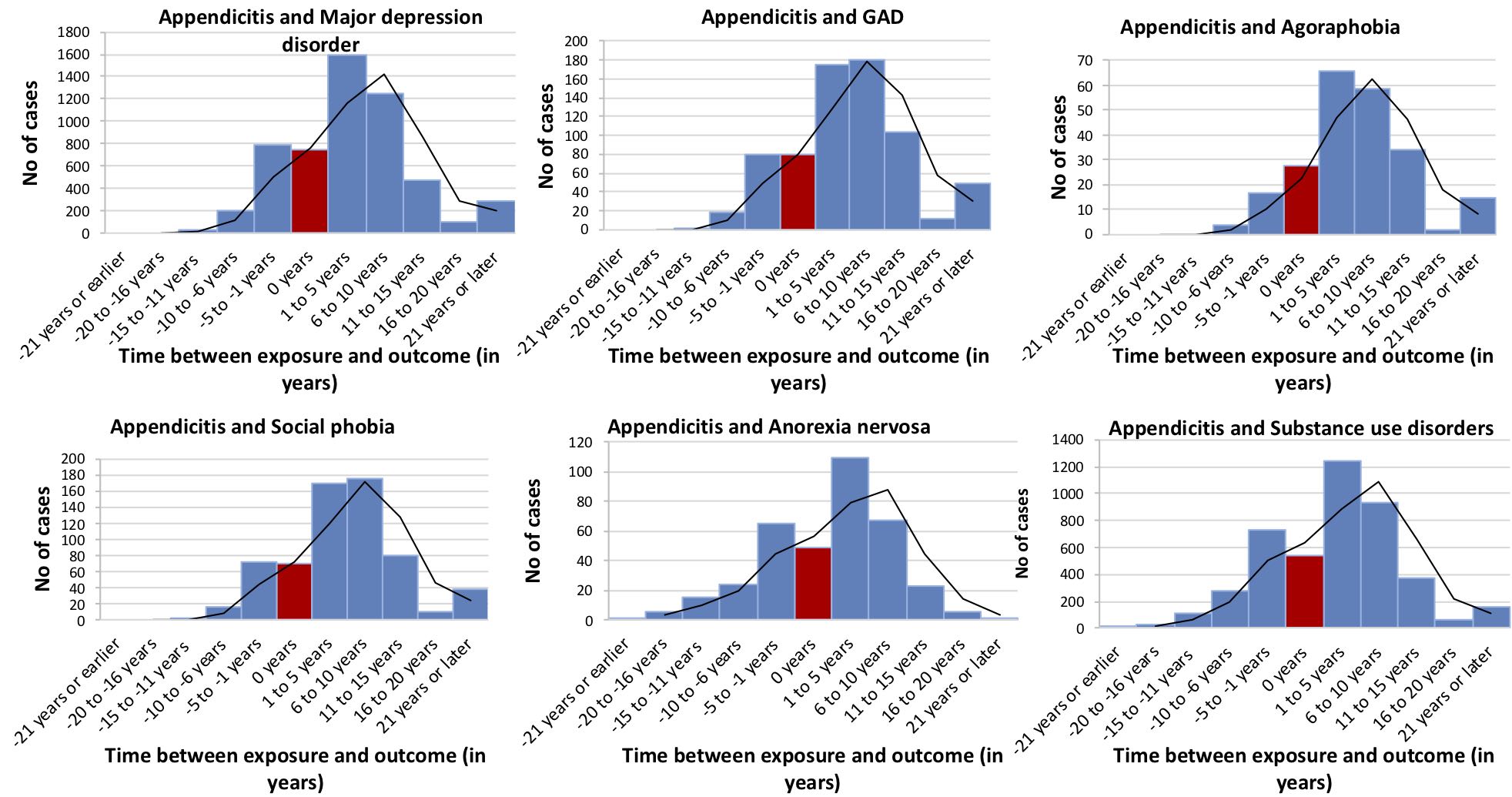


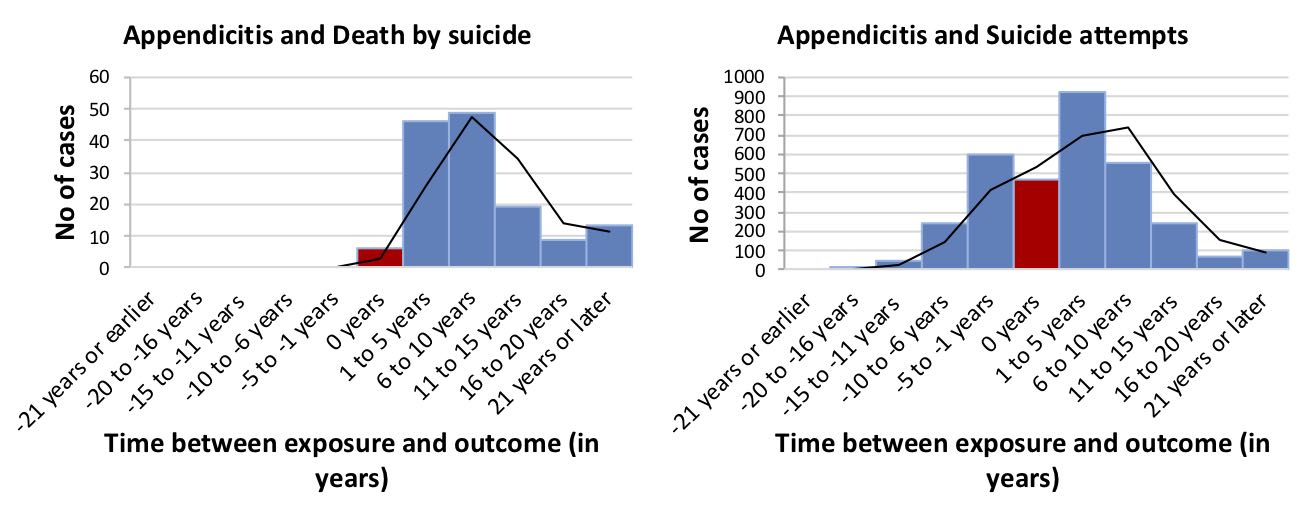


*Note:* Negative values represent the situation when register-based records of psychiatric disorder and/or suicidal behavior preceded in time the record of tonsillectomy and acute appendicitis (i.e., age of outcome is lower than that of exposure), while positive values represent the opposite situation. A value ‘zero’ represents individuals for whom the age at exposure was equal to age of outcome (presented by red bar to ease visualization). A trend line represents a moving average.

Abbreviation: GAD, Generalized anxiety disorder

**S References**

1 Rück C, Larsson KJ, Lind K, Perez-Vigil A, Isomura K, Sariaslan A *et al.* Validity and reliability of chronic tic disorder and obsessive-compulsive disorder diagnoses in the Swedish National Patient Register. *BMJ Open* 2015; **5**: e007520.

2 Larsson H, Rydén E, Boman M, Långström N, Lichtenstein P, Landén M. Risk of bipolar disorder and schizophrenia in relatives of people with attention-deficit hyperactivity disorder. *Br J Psychiatry J Ment Sci* 2013; **203**: 103–106.

3 Runeson B, Tidemalm D, Dahlin M, Lichtenstein P, Långström N. Method of attempted suicide as predictor of subsequent successful suicide: national long term cohort study. *BMJ* 2010; **341**: c3222.

4 Fernández de la Cruz L, Rydell M, Runeson B, D’Onofrio BM, Brander G, Rück C *et al.* Suicide in obsessive-compulsive disorder: a population-based study of 36 788 Swedish patients. *Mol Psychiatry* 2016. doi:10.1038/mp.2016.115.
